# 肺癌家系肿瘤风险度病例对照研究与预测模型

**DOI:** 10.3779/j.issn.1009-3419.2011.07.04

**Published:** 2011-07-20

**Authors:** 欢 林, 文昭 钟, 学宁 杨, 红虹 严, 一龙 吴

**Affiliations:** 510080 广州，广东省肺癌研究所，广东省人民医院，广东省医学科学院 Guangdong Lung Cancer Institute, Guangdong General Hospital, Guangdong Academy of Medical Sciences, Guangzhou 510080, China

**Keywords:** 遗传流行病学, 家族聚集, 一级亲属, 肺肿瘤, 遗传, Genetic epidemiology, Familial aggregation, First-degree relative, Lung neoplasms, Inheritance

## Abstract

**背景与目的:**

对人群每年进行低剂量胸部CT筛查可提高早期肺癌诊断率，但其假阳性率较高，常导致不必要的手术。本研究拟建立肺癌家系风险度预测模型，从中细分高危人群，从而提高筛选效能。

**方法:**

以经病理确诊的肺癌患者的家系作为研究人群，同时收集先证者的配偶家系作为对照家系，共收集先证者家系633例和对照家系565例。应用SPSS 17.0进行统计学分析。

**结果:**

先证者家系一级亲属患肿瘤的风险性为对照组家系一级亲属的1.71倍（OR=1.71, *P* < 0.001）。家系中患癌个数分别为=1和≥2的两组与对照组比较有统计学差异（*P*=0.005, *P*=0.002）。建立回归模型后赋值得到与普通人群相比的肺癌风险度为0.38-63.08（倍）。风险度为普通人群10倍以上的群体，应用本模型的正确率为88.1%。

**结论:**

如果一级亲属患癌个数越多，患肺癌的风险越高。根据本研究建立的风险度预测模型，风险度达普通人群10倍以上的主要为重度吸烟的吸烟人群，应加强筛查。特点为：有肺部既往疾病史的重度吸烟人群，加上男性、职业暴露和一级亲属肿瘤家族史三项中的任一项；有肺部既往疾病史或重度吸烟的人群中，有职业暴露的男性且一级亲属有不少于两位肿瘤患者。

## Abstract (en)

**Background and objective:**

Annual lung screening using spiral computed tomography (CT), has a high sensitivity of detecting early lung cancer (LC), but its high rates of false-positive ofen lead to unnecessary surgery. The aim of this study is to create a forecasting model of high risk individuals to lung cancer.

**Methods:**

The pathologic diagnoses of LC in Guangdong Lung Cancer Institute were consecutively chosen as the probands. All the members of the frst-degree relatives of probands' and their spouses' were enrolled in this study. Tese pedigrees consisted of 633 probands' pedigrees and 565 spouses' pedigrees. Unless otherwise stated, analyses were performed using the SPSS 17.0 statistical sofware package.

**Results:**

Compared with the control, a family history of carcinoma in frst-degree relatives was signifcantly associated with LC risk (OR=1.71, *P* < 0.001), the sub-group of either one infected individual or more than two infected individuals in frst-degree relatives showed signifcantly statistical differences (*P*=0.005, *P*=0.002). In the forecasting model, the risk compared to that in Chinese population was from 0.38 to 63.08 folds. In the population whose risk was more than 10 times to the Chinese population, the accuracy rate of prediction was 88.1%.

**Conclusion:**

A family history of carcinoma in frst-degree relatives was signifcantly associated with increased LC risk. The more infected individuals exist in frst-degree relatives, the more risk was showed. In the forecasting model, smokers especially heavy ones whose risk were more than 10 times to the Chinese population should be receive annual screening. The population are positive at least any two conditions which including male, lung disease history, occupation expose and history of cancer in frst-degree relative.

大多数人类肿瘤和环境因素相关，但同样暴露于特定致癌物，却仅部分人群发病。另外，某些肿瘤也有明显的家族聚集现象。可见，除环境因素外，遗传背景尤其是基因的多态性差异也是重要的决定因素。来自冰岛等地的研究揭示，家族聚集可表现为不同类型肿瘤的聚集，提示存在共同的遗传因素。例如，雌激素相关基因可能和经产妇患乳腺癌与肺癌风险性增加存在交叉联系^[1-4]^。瑞典的研究^[5]^揭示，吸烟可能导致胰腺癌与肺癌的家族聚集，而胰腺癌与乳腺癌的家族聚集可能与BRCA2的遗传变异有关。本文通过对单位时间内的肺癌患者连续性收集调查资料，进行大样本量遗传流行病学调查，对肺癌患者的肿瘤家族聚集性进行研究，并建立肺癌风险度预测模型，期望有助于高危人群的筛选和早期发现。

## 1 材料与方法

### 1.1 病例选择

连续性收集2009年10月-2010年12月于广东省肺癌研究所病理学确诊的肺癌患者，并以其作为先证者，其所在家系确定为先证者家系，其一级亲属（被调查者的父母、子女及同胞）为家系成员。因肺癌先证者的子女发病率极低，与大部分年龄未到发病高峰期有关，且绝大部分子女为先证者和配偶对照组的共同子女，因此统计时将子女从一级亲属范围剔除。

### 1.2 对照选择

对照组为肺癌先证者的配偶家系，纳入研究的配偶无肿瘤史，与肺癌家系成员之间不存在任何血缘关系。由同一调查员采用相应调查表记录对照家系成员的一般情况及家系资料，另一调查员进行复核。

### 1.3 材料收集

由调查员对初治的肺癌患者进行面访，在先证者本人或其亲属签定知情同意书后，应用统一的调查表，对肺癌先证者及其配偶进行调查。调查表内容包括性别、年龄分组、吸烟指数、肺部既往疾病史、居住环境、职业接触、一级亲属肿瘤家族史和亲属的情况等。由最了解情况者作为问讯对象，提高可靠性。为了减少回忆偏倚，我们尽可能增加了样本量，同时又让调查对象对一些不确定情况通过电话咨询的方式进行证实。

### 1.4 统计分析

用EpiData 3.1软件建立数据库，应用SPSS 17.0对先证者及其家系资料和对照资料进行统计学分析。分类资料的比较采用卡方检验，如不满足卡方检验条件者采用*Fisher Exact*检验；计量资料的比较采用两独立样本*t*检验。所有统计均采用双侧检验，检验水准为0.05。Crude OR通过卡方检验计算得出，Adjusted OR通过*Logistic*回归分析得到。*Logistic*逐步回归模型对因素的筛选条件为进入标准*P* < 0.05，剔除标准为*P* > 0.10。

## 2 结果

### 2.1 均衡性检验

先证者及对照组的籍贯属于中国东部沿海的25个省或直辖市，广东省内患者来自广东省21个地级市。两者家系在年龄、地区、居住环境和一级亲属人数的比较，无统计学差异（表 1）。先证者的肺癌分期采用UICC的TNM2009分期，其中Ⅰ期患者96例，Ⅱ期患者63例，Ⅲ期患者157例，Ⅳ期患者317例。

**1:** 临床流行病学资料比较 Comparison of epidemiology data

Characteristic	Case (*n* =633) [*n* (%)]	Control (*n* =565) [*n* (%)]	*P*
Age cohort			0.070
50 years	125 (19.7)	136 (24.1)	
≥50 years	508 (80.3)	429 (75.9)	
Province^a^			0.550
Guangdong Province	461 (72.8)	402 (71.3)	
Non-Guangdong Province	172 (27.2)	162 (28.7)	
Daily contact			0.374
No	527 (83.3)	481 (85.1)	
Yes	106 (16.7)	84 (14.9)	
^a^: Missing values were not included in percentage calculations.			

### 2.2 先证者与配偶年龄分布

由图 1可见，先证者由52岁开始迅速上升，56岁左右达高峰，至80岁后迅速下降，大致呈单峰分布。而配偶的年龄分布大致与之匹配。

**1:**
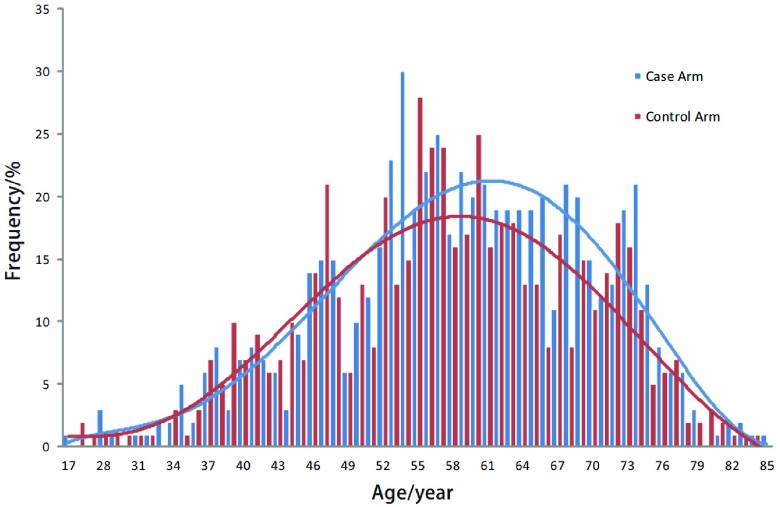
病例组和对照组年龄分布图 The distribution of age in case arm and control arm

### 2.3 一级亲属患癌风险

由表 2、表 3可见，肺癌患者一级亲属的患癌风险性明显高于对照组。家系患癌个数分别为0、1和≥2三组，有统计学差异，发病年龄分层分析显示晚发肺癌差异较早发肺癌明显，但早发肺癌样本量相对较少。

**2:** 先证者一级亲属患癌风险 Risk of cancer in first-degree relatives of proband

Factor	Case/Control	Crude OR (95%CI)	Adjusted ORa (95%CI)
Proband’s family history			
No	432/438	1.00	1.00
Yes	201/127	1.60 (1.24-2.08)	1.71 (1.28-2.28)
N of families with			
0	432/438	1.00	1.00
1	149/111	1.36 (1.03-1.80)	1.55 (1.14-2.12)
2+cancers	52/16	3.30 (1.85-5.86)	2.65 (1.42-4.94)
^a^: ORs calculated by conditional logistic regression adjusted for sex, lung disease history, smoking status, living environment and occupation expose.			

**3:** 先证者的一级亲属患癌风险性（以年龄分层） Risk of cancer in first-degree relatives of Proband stratified by age

Age cohort	Factors		Case/Control	Crude OR (95%CI)	Adjusted OR^a^ (95%CI)
< 50 years	Proband’s family history	No	86/112	1.00	1.00
		Yes	39/24	2.12 (1.18-3.78)	2.01 (1.08-3.74)
	N of families with	0	86/112	1.00	1.00
		1	32/23	1.81 (0.99-3.32)	1.71 (0.90-3.28)
		2+cancers	7/1	9.12 (1.10-75.50)	8.85 (1.01-77.80)
≥50 years	Proband’s family history	No	346/326	1.00	1.00
		Yes	162/103	1.48 (1.11-1.98)	1.64 (1.18-2.27)
	N of families with	0	346/326	1.00	1.00
		1	117/88	1.25 (0.91-1.72)	1.52 (1.07-2.17)
		2+cancers	45/15	2.83 (1.55-5.17)	2.21 (1.14-4.28)
^a^: ORs calculated by conditional logistic regression adjusted for sex, lung disease history, smoking status, living environment and occupation expose.					

### 2.4 肺癌风险度的判别预测模型

为控制混杂因素，提高预测准确率，建立以性别（女、男）^[6]^、吸烟指数（0、 < 400和≥400）^[7]^、肺部既往疾病史（无、有）^[8]^、生活接触史（无、有）^[9]^、职业接触史（无、有）^[10]^等公认的危险因素和本研究证实的一级亲属患癌个数（0、1、≥2），年龄分组（< 50岁、≥50岁）^[11]^等肺癌风险度影响因素为自变量（各变量的取值以第一种情况为0，余各情况依次递增），是否为先证者为因变量建立二分类*Logistic*前进法逐步回归模型。最终保留在模型中的自变量为性别、吸烟指数、肺部既往疾病史、职业接触史和一级亲属患癌个数（表 4）。

**4:** 预测模型的二分类*Logistic*回归分析模型 A binary *Logistic* regression analysis of forecasting model

Variable	B	SE	Wald	*P*	OR (95%CI)
Sex	0.464	0.178	6.78	0.009	1.59 (1.12-2.26)
Smoking status			62.69	< 0.001	
Light smoker	0.135	0.241	0.31	0.576	1.14 (0.71-1.84)
Heavy smoker	1.545	0.211	53.74	< 0.001	4.69 (3.10-7.09)
Lung disease history	1.689	0.322	27.49	< 0.001	5.41 (2.88-10.18)
Occupation expose	0.470	0.172	7.44	0.006	1.60 (1.14-2.24)
N of families with			15.02	0.001	
1	0.436	0.158	7.63	0.006	1.55 (1.14-2.11)
2+cancers	0.957	0.318	9.06	0.003	2.60 (1.40-4.86)
Constant a′	-0.867	0.102	72.03	< 0.001	0.420

根据表 4得到回归函数logit(P)=-0.867+0.464^*^性别+0.135^*^吸烟指数1+1.545^*^吸烟指数2+1.689^*^肺部既往疾病史+0.470^*^职业接触+0.436^*^一级亲属患癌个数1+0.957^*^一级亲属患癌个数2。

① 模型用于预测：由于病例对照研究的非条件*Logistic*回归得不到常数项a′的估计值，不能直接用于预测，需要对常数项进行校正，即：

$
{\rm{a = a' - ln(}}\frac{{{n}{\rm{1}}{q0}}}{{n0q1}}{\rm{)}}
$

其中*n*1和*n*0分别为病例和对照的样本含量，*q*1和*q*0为特定人群中发病和不发病的先验概率。以《中国肿瘤登记地区2006年肿瘤发病和死亡资料分析》中肺癌发病率49.7/10万用于常数项的校正^[12]^，然后再用调整后的*α*作为*Logistic*回归方程的常数项计算预测的肺癌发病概率（表 5）。

**5:** 研究对象的肺癌发病概率预测及相对风险度 Prediction of lung cancer morbidity and relative risk in the study objects

Row	Sex	Lung disease history	Occupation expose	Light smokers	Heavy smokers	Infected individual (=1)	Infected individual (≥2)	Constant	Probability	RR^a^
	0.464	1.689	0.47	0.135	1.545	0.436	0.957	-0.867		
1	0	0	0	0	0	0	0		0.000, 186	0.38
2	0	0	0	1	0	0	0		0.000, 213	0.43
3	0	0	0	0	0	1	0		0.000, 288	0.58
4	1	0	0	0	0	0	0		0.000, 296	0.60
5	0	0	1	0	0	0	0		0.000, 298	0.60
6	0	0	0	1	0	1	0		0.000, 330	0.66
7	1	0	0	1	0	0	0		0.000, 339	0.68
8	0	0	1	1	0	0	0		0.000, 341	0.69
9	1	0	1	0	0	0	0		0.000, 474	0.95
10	0	0	0	0	0	0	1		0.000, 485	0.98
11	0	0	1	1	0	1	0		0.000, 528	1.06
12	1	0	0	1	0	1	0		0.000, 525	1.06
13	1	0	1	1	0	0	0		0.000, 543	1.09
14	0	0	0	1	0	0	1		0.000, 556	1.12
15	1	0	1	1	0	1	0		0.000, 840	1.69
16	0	0	0	0	1	0	0		0.000, 874	1.76
17	1	0	0	1	0	0	1		0.000, 884	1.78
18	0	0	1	1	0	0	1		0.000, 889	1.79
19	0	1	0	0	0	0	0		0.001, 009	2.03
20	0	1	0	1	0	0	0		0.001, 155	2.32
21	0	0	0	0	1	1	0		0.001, 351	2.72
22	1	0	0	0	1	0	0		0.001, 390	2.80
23	0	0	1	0	1	0	0		0.001, 398	2.81
24	1	0	1	1	0	0	1		0.001, 414	2.84
25	1	1	0	0	0	0	0		0.001, 605	3.23
26	0	1	1	0	0	0	0		0.001, 615	3.25
27	0	1	0	1	0	1	0		0.001, 786	3.59
28	1	1	0	1	0	0	0		0.001, 837	3.70
29	0	1	1	1	0	0	0		0.001, 848	3.72
30	1	0	0	0	1	1	0		0.002, 149	4.32
31	0	0	1	0	1	1	0		0.002, 162	4.35
32	1	0	1	0	1	0	0		0.002, 224	4.47
33	0	0	0	0	1	0	1		0.002, 275	4.58
34	1	1	1	0	0	0	0		0.002, 568	5.17
35	1	1	0	1	0	1	0		0.002, 841	5.72
36	0	1	1	1	0	1	0		0.002, 858	5.75
37	1	1	1	1	0	0	0		0.002, 939	5.91
38	0	1	0	1	0	0	1		0.003, 008	6.05
39	1	0	1	0	1	1	0		0.003, 439	6.92
40	1	0	0	0	1	0	1		0.003, 619	7.28
41	0	0	1	0	1	0	1		0.003, 641	7.33
42	1	1	1	1	0	1	0		0.004, 546	9.15
43	0	1	0	0	1	0	0		0.004, 731	9.52
44	1	1	0	1	0	0	1		0.004, 784	9.62
45	0	1	1	1	0	0	1		0.004, 812	9.68
46	1	0	1	0	1	0	1		0.005, 790	11.65
47	0	1	0	0	1	1	0		0.007, 317	14.72
48	1	1	0	0	1	0	0		0.007, 525	15.14
49	0	1	1	0	1	0	0		0.007, 570	15.23
50	1	1	1	1	0	0	1		0.007, 654	15.40
51	1	1	0	0	1	1	0		0.011, 637	23.41
52	0	1	1	0	1	1	0		0.011, 707	23.56
53	1	1	1	0	1	0	0		0.012, 039	24.22
54	0	1	0	0	1	0	1		0.012, 320	24.79
55	1	1	1	0	1	1	0		0.018, 619	37.46
56	1	1	0	0	1	0	1		0.019, 593	39.42
57	0	1	1	0	1	0	1		0.019, 711	39.66
58	1	1	1	0	1	0	1		0.031, 349	63.08
^a^: prediction of study object with lung cancer morbidity compared to Chinese population.										

由表 5可见，模型将本研究的病例组和对照组分为58个亚组，并列出与普通人群肺癌发病率比较得到的相对风险度。根据第四版流行病学教科书关于暴露与疾病联系强度的描述，RR在1.0-1.1为无联系，RR在1.2-1.4代表联系强度为弱，RR为1.5-2.9代表联系强度为中等，RR为3.0-9.0代表联系强度为强，RR≥10代表联系强度为很强。表 5中风险度为普通人群10倍以上的群体共13个亚组，该人群主要为重度吸烟的吸烟人群，在性别、肺部既往疾病史、职业接触史和一级亲属肿瘤家族史中具备至少两个以上阳性。

② 模型用于判别以验证正确率（表 6）：根据估计概率进行判别归类，第一类为非肺癌（对照），第二类为肺癌（病例）。如果估计概率 < 0.5，则将其判定为第一类；如果估计概率 > 0.5，则将其判定为第二类；如果=0.5，暂不归类。最后将结果与实际情况对照，得到模型的正确率。

**6:** 预测模型效果检验 Classification table^a^ of forecasting model

Observed group	Predicted group		Percentage correct (%)
	Control	Case	
Control	440	125	77.9
Case	232	401	63.3
Total	672	526	70.2
^a^: The cut value is 0.500.			

③ 风险度为普通人群10倍以上的群体预测正确率（表 7，表 8）由表 7和表 8可见，在该群体应用本预测模型的正确率达到了88.1%，有良好的应用价值。

**7:** 风险度为普通人群十倍以上的群体预测情况 Prediction of people whose degree of risk are more than ten times to the Chinese population

	Probability	RR	*n*	Case/control (Forecast)	Case/control (Fact)
46	0.005, 790	11.65	20	20/0	17/3
47	0.007, 317	14.72	0	0	0
48	0.007, 525	15.14	0	0	0
49	0.007, 570	15.23	2	2/0	2/0
50	0.007, 654	15.40	0	0	0
51	0.011, 637	23.41	0	0	0
52	0.011, 707	23.56	0	0	0
53	0.012, 039	24.22	26	26/0	22/4
54	0.012, 320	24.79	0	0	0
55	0.018, 619	37.46	8	8/0	8/0
56	0.019, 593	39.42	0	0	0
57	0.019, 711	39.66	0	0	0
58	0.031, 349	63.08	3	3/0	3/0

**8:** 预测模型中风险度为普通人群十倍以上的群体效果检测 Classification table^a^ of people whose degree of risk are more than ten times to the Chinese population in the forecasting model

Observed group	Predicted group		Percentage correct (%)
	Control	Case	
Control	0	7	0
Case	0	52	100
Total	0	59	88.1
^a^: The cut value is 0.500.			

## 3 讨论

吸烟是目前肺癌最重要的危险因素。其它的危险因素包括二手烟、生活接触、职业暴露、HPV等病毒感染、空气污染和结核等。基因易感性在年轻肺癌中起到尤其重要的意义。

肿瘤二次打击学说主要适用于有遗传倾向的肿瘤，如视网膜母细胞瘤等，对应的临床特点为：早年发病、病灶双侧或多发和家族聚集倾向，其中家族史是最明显的临床特征。Xu等^[13]^收集了1, 561例肺癌先证者的12, 817例一级亲属的资料进行分析，提示孟德尔衰减模型和共显性模型均能容纳肺癌的病因解释，而当把发病年龄分布纳入模型时，则发现多基因和环境因子的交互作用模型更符合总体人群的肺癌发病分布。

本研究为单位时间内连续收集病例的大样本量病例对照研究，地域分布均衡，具有人群普遍性。Ziogas等^[14]^研究表明，以人口登记为基础的研究其家族史假阳性率较高，以临床患者为基础的研究比较可靠。本研究的调查对象为临床患者，风险比为控制性别、年龄分组、肺部既往疾病史、吸烟指数、居住环境、职业接触得到的调整OR，因此，与以人群为基础的研究比较可信度较高。

美国肺癌遗传流行病学联盟2004年首次定位了和肺癌家系关联的区域——染色体6q23-25，并发现随着家系中癌症成员的增加，易感基因与6号染色体上的遗传标记的连锁相关性也增强^[15]^。本文发现在调整性别、年龄分组、肺部既往疾病史、吸烟指数、生活接触和职业接触后，肺癌患者一级亲属的患癌风险性明显高于对照组，且家系患癌个数为1和≥2的两个亚组均有统计学差异（OR=1.55, *P*=0.005; OR=2.65, *P*=0.002），结论与前述类似。这提示随着家系中肿瘤患者的增加，体现的肿瘤遗传易感性强度有增加的趋势，这也是将家系中一级亲属患癌个数列为肿瘤风险度因素之一的依据。另外，发病年龄分层分析显示晚发肺癌差异较早发肺癌明显，除早发肺癌例数相对较少的因素外，也可能与肺癌由低肿瘤易感性的遗传多态性决定有关。

日本有一项大规模的前瞻性队列研究JPHC研究^[16]^表明所有癌症家族史与肺癌发病风险增加无关。作为前瞻性研究，其与本研究肺癌先证者一级亲属患癌风险性有高度统计学意义的结论相反。从肺癌与其它肿瘤的家族聚集现象证明有共同遗传因素影响的众多研究，以及肺癌发病的理论推断，肺癌先证者的肿瘤家族史对肺癌风险性的提高应有影响，但影响低于肺癌家族史。分析该研究的随访，发现该队列研究入组132, 972受试者，年龄40岁-69岁，随访102, 255例，随访13年发现791例新发肺癌。在基线记录资料后，追踪新发肺癌患者资料而未更新肿瘤家族史资料。而本研究以临床患者为目标人群，即时记录对应的肿瘤家族史，目的性和时效性强。因此，JPHC研究的样本量基数大，肿瘤家族史资料未更新，可能导致关联关系被掩盖。

早期肺癌筛查国际行动计划（International Early Lung Cancer Action Program, I-ELCAP）此前的研究数据^[17]^表明，每年进行低剂量CT筛查可检查出Ⅰ期肺癌，Ⅰ期肺癌患者若立即进行手术切除肿瘤，其10年生存率可达92%，而所有未治疗的Ⅰ期患者将在5年内死亡。该研究表明低剂量CT筛查可增加早期肺癌的诊断率，从而使患者获得较好的生存结果。但该筛查的假阳性率一直被诟病。美国国立卫生研究院（National Institutes of Health, NIH）一项大样本、长期随机临床研究^[18]^表明，对高危人群（吸烟或曾经吸烟达每年30包以上，年龄55岁-74岁）进行低剂量CT扫描筛查肺癌的假阳性率较高，常常因“错误预警”导致不必要的检查、活检和手术。因此，建立肺癌风险度模型，综合评估肺癌发病的各个危险因素，找到真正的肺癌高危人群，是性价比最高的途径。

本文针对吸烟指数、性别、年龄分组、一级亲属患癌个数、肺部既往疾病史、生活接触史和职业接触史，建立回归模型，赋值后得到各亚组肺癌发病概率与人群相比的风险度在0.38-63.08的结论，准确率为70.2%。这可能是因为在低风险的群体，暴露因素和疾病联系的强度不大从而影响了预测效率。而在联系强度为很强、风险度为普通人群10倍以上的群体，应用本模型的预测准确率为88.1%。特点为：有肺部既往疾病史的重度吸烟人群，加上男性、职业暴露和一级亲属肿瘤家族史三项中的任一项；有肺部既往疾病史或重度吸烟的人群中，有职业暴露的男性且一级亲属有不少于两位肿瘤患者。因此，建议风险度为人群10倍以上的高危人群可每年进行低剂量CT筛查，可望提高筛查效能。但因病例组和对照组为配偶关系，生活环境基本相同，所以生活接触史未保留在模型中，应用时应结合本因素综合考虑。
